# Kaempferol: A Key Emphasis to Its Anticancer Potential

**DOI:** 10.3390/molecules24122277

**Published:** 2019-06-19

**Authors:** Muhammad Imran, Bahare Salehi, Javad Sharifi-Rad, Tanweer Aslam Gondal, Farhan Saeed, Ali Imran, Muhammad Shahbaz, Patrick Valere Tsouh Fokou, Muhammad Umair Arshad, Haroon Khan, Susana G. Guerreiro, Natália Martins, Leticia M. Estevinho

**Affiliations:** 1 University Institute of Diet and Nutritional Sciences, Faculty of Allied Health Sciences, The University of Lahore, Lahore 54000, Pakistan; mic_1661@yahoo.com; 2 Student Research Committee, School of Medicine, Bam University of Medical Sciences, Bam 44340847, Iran; 3 Zabol Medicinal Plants Research Center, Zabol University of Medical Sciences, Zabol 61615-585, Iran; 4 School of Exercise and Nutrition, Deakin University, Victoria 3221, Australia; tgondal@deakin.edu.au; 5 Department of Food Science, Nutrition & Home Economics, Institute of Home and Food Sciences, Government College University, Faisalabad 38000, Pakistan; f.saeed@gcuf.edu.pk (F.S.); aliimran.ft@gmail.com (A.I.); umairfood1@gmail.com (M.U.A.); 6 Department of Food Science and Technology, MNS-University of Agriculture, Multan 66000, Pakistan; shahbaz.ft@mnsuam.edu.pk; 7 Department of Biochemistry, Faculty of Science, University of Yaounde 1, Yaounde P.O. Box 812, Cameroon; 8 Department of Pharmacy, Faculty of Chemical & Life Sciences, Abdul Wali Khan University Mardan, Mardan 23200, Pakistan; hkdr2006@gmail.com; 9 Faculty of Medicine, University of Porto, Alameda Prof. Hernâni Monteiro, 4200-319 Porto, Portugal; sguerreiro@ipatimup.pt; 10 Institute for Research and Innovation in Health (i3S), University of Porto, 4200-135 Porto, Portugal; 11 Faculty of Nutrition and Food Science, University of Porto, 4200-465 Porto, Portugal; 12 Department of Biology and Biotechnology, School of Agriculture of the Polytechnic Institute of Bragança (ESA-IPB), Campus de Santa Apolónia, 5301-854 Bragança, Portugal; 13 CIMO, Mountain Research Center, Polytechnic Institute of Bragança. Campus Santa Apolónia, 5301-855 Bragança, Portugal

**Keywords:** kaempferol, pharmacokinetics, pharmacodynamics, antioxidant, anticancer, chemoprevention, apoptosis, cell cycle arrest, metastasis, reactive oxygen species

## Abstract

A marked decrease in human cancers, including breast cancer, bone cancer, and cervical cancer, has been linked to the consumption of vegetable and fruit, and the corresponding chemoprotective effect has been associated with the presence of several active molecules, such as kaempferol. Kaempferol is a major flavonoid aglycone found in many natural products, such as beans, bee pollen, broccoli, cabbage, capers, cauliflower, chia seeds, chives, cumin, moringa leaves, endive, fennel, and garlic. Kaempferol displays several pharmacological properties, among them antimicrobial, anti-inflammatory, antioxidant, antitumor, cardioprotective, neuroprotective, and antidiabetic activities, and is being applied in cancer chemotherapy. Specifically, kaempferol-rich food has been linked to a decrease in the risk of developing some types of cancers, including skin, liver, and colon. The mechanisms of action include apoptosis, cell cycle arrest at the G2/M phase, downregulation of epithelial-mesenchymal transition (EMT)-related markers, and phosphoinositide 3-kinase/protein kinase B signaling pathways. In this sense, this article reviews data from experimental studies that investigated the links between kaempferol and kaempferol-rich food intake and cancer prevention. Even though growing evidence supports the use of kaempferol for cancer prevention, further preclinical and clinical investigations using kaempferol or kaempferol-rich foods are of pivotal importance before any public health recommendation or formulation using kaempferol.

## 1. Introduction

Kaempferol represents one of the most encountered aglycone flavonoids in the form of glycoside. It is a tetrahydroxyflavone in which the four hydroxy groups are located at positions 3, 5, 7, and 4′, and it is a yellow compound [1]. Kaempferol is found in various plant parts, such as seeds, leaves, fruits, flowers, and even vegetables [2,3,4]. Kaempferol and its glycosylated derivatives have been shown to be cardioprotective, neuroprotective, anti-inflammatory, antidiabetic, antioxidant, antimicrobial, antitumor, and have anticancer activities [5].

Epidemiological studies showed that a high intake of kaempferol is associated with decreased incidence of different types of cancer, among which cancer in organs like skin, liver, colon, ovary, pancreas, stomach, and bladder [6,7]. In this context, kaempferol consumption and related application in cancer therapy are gaining huge attention among the research community [6,7,8]. The cancer prevention is mostly achieved by inhibiting the proliferation of cancer cells through increasing the apoptosis [9,10,11]. Indeed, kaempferol inhibits various cancer cells by triggering apoptosis, cell cycle arrest at G2/M phase, downregulation of signaling pathways and phosphoinositide 3-kinase (PI3K)/protein kinase B (AKT), expression of epithelial-mesenchymal transition (EMT)-related markers (N-cadherin, E-cadherin, Snail, and Slug), and matrix metallopeptidase 2 (MMP-2), metastasis-related markers [12,13]. Kaempferol also induces the activation of cysteine proteases involved in apoptosis initiation and execution, caspases-3, -7, -9, and Poly (ADP-ribose) polymerase (PARP) [14], therefore preventing the accumulation of reactive oxygen species (ROS) involved in cancer development [15]. The inhibition of angiogenesis was also reported as well as the capacity of kaempferol to preserve normal cell viability [15]. In this context, this review summarizes data on pharmacodynamics, chemopreventive and anticancer effects, and mechanisms of action of kaempferol.

## 2. Metabolism and Pharmacokinetics of Kaempferol

Studies on the in vitro and in vivo pharmacokinetics of kaempferol commonly ingested as high polarity glycosides revealed that this polyphenol is poorly absorbed compared to the aglycones with intermediate polarity [16].

Kaempferol lipophilicity allowed its absorption in the small intestine through passive and facilitated diffusion or active transport [17]. Of note, intake of 14.97 mg kaempferol/day and 27 mg kaempferol from tea resulted in a plasma concentration of 16.69 ng/mL and 15 ng/mL, respectively [18]. The absorbed kaempferol undergoes metabolic transformation to yield the glucuronides and sulfoconjugates forms in the liver [19] and small intestine by intestinal conjugation enzymes [17]. As well, kaempferol and its glycosides are metabolized in the colon by the bacterial microflora that releases the aglycones and broke aglycone C3 ring to form compounds such as 4-methylphenol, phloroglucinol, and 4-hydroxyphenylacetic acid, that are either absorbed and can reach systemic circulation and tissues or be excreted in feces and urine [20,21,22,23,24,25,26,27]. To overcome the low bioavailability of kaempferol, its combination with quercetin increase its bioavailability, consequently improving its bio-efficacy. In fact, studies prove that nanoformulations (e.g., nanoparticles, nanoemulsions, nanoencapsulation) containing kaempferol will be extremely beneficial in improving their bioavailability and consequent efficacy and selectivity for mutated cells, while their effect on normal cells will be limited [28]. Indeed, kaempferol exerts protective effects in non-mutated cells, whereas it triggers apoptosis in those mutated ones. These aspects are mostly linked to the remarkable antioxidant effects of kaempferol, namely acting directly in antioxidant enzymes, capable of efficiently inhibit ROS generation and lipid peroxidation, and, finally, preventing the occurrence of cell damages, in a broad-spectrum activity [29].

## 3. Antioxidant Potential of Kaempferol

**Figure 1:**
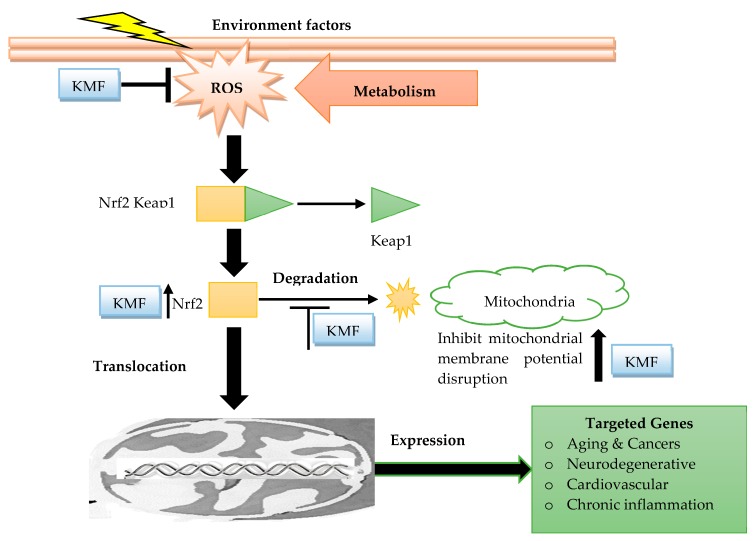
Antioxidant mechanisms of action of kaempferol: The kaempferol reduces the ROS metabolism, cleavage of anti-inflammatory membranes, and disrupts their molecular mechanism as a mechanistic concern to tackle cancer-related expressions (KMF: Kaempferol; Nrf2: Nuclear factor erythroid 2-related factor 2; Keap1: Kelch-like ECH-associated protein 1; RO: Reactive oxygen species).

Kaempferol and its glycosides, as well as kaempferol-containing plants, portray antioxidant potency both in culture and in animal models [26,27], and it has the capacity to decrease the production of free radicals and other products like reactive oxygen species (ROS). ROS are aerobic metabolism by-products that can induce malignant cell transformation. Thereafter, ROS production inhibition can reverse malignant cancer cell phenotype [28,29,30,31]. Usually, superoxide anion is either converted by superoxide dismutase into H_2_O_2_ that react with reduced metals (e.g., ferrous or cuprous ions), to yield the highly reactive hydroxyl radical or form peroxynitrite by reacting with nitric oxide. These two highly reactive species, hydroxyl radical and peroxynitrite, can cause lipids, proteins, or DNA damages [32]. At submicromolar concentrations, kaempferol is not only a potent scavenger of superoxide anion, hydroxyl radical, and peroxynitrite [32,33,34], but it also inhibits pro-oxidant enzymes, such as xanthine oxidase [35], and activates antioxidant enzymes such as superoxide dismutase, catalase, and heme oxygenase-1 (Figure 1) [36,37] and even prevents the generation of hydroxyl radical by chelating cuprous or ferrous [38,39]. Also, and not least important to highlight, is that kaempferol contains hydroxyl groups at C3, C5, and C4, an oxo group at C4, and a double bond at C2-C3 that might explain its antioxidant activity [32].

Conclusively, kaempferol can control the cancer through its antioxidative/antinitrosative and anti-inflammatory potential by restoring the cell redox hemostasis by inhibiting the NF-κB pathway and to up-regulate the Nrf2 transcriptional pathway (Figure 1).

## 4. Anticancer Properties of Kaempferol

### 4.1. Anti-Breast Cancer Activity

**Figure 2:**
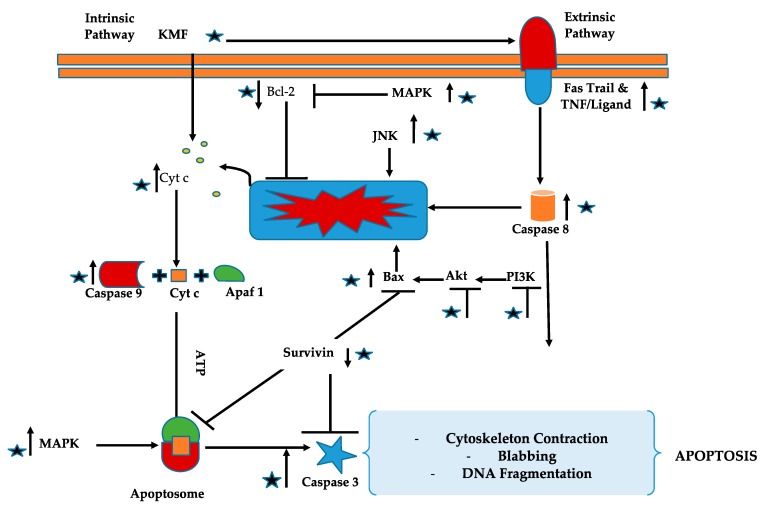
Anticancer role of kaempferol: Mechanistically, it can induce anticancer effects mainly through downregulation of the expressions of proteins involved in the cancer progression and formation alongside apoptosis induction, cell cycle arrest, and decreasing the expression for anti-inflammatory proteins.

Breast cancer burden has been increasing over the years and it represents the most-encountered cancer in women [40]. At micromolar concentrations, kaempferol effectively inhibits the growth of breast cancer cell lines (VM7Luc4E2, MDA- MB-231, MCF-7) [40,41,42,43]. Also, kaempferol markedly inhibits the bisphenol A (BPA) (endocrine-disrupting chemicals) and triclosan (TCS)-induced anti-apoptotic processes [44], causes cell arrest at the G2/M stage, and even induces apoptosis and DNA fragmentation at the sub-G0 phase (Table 1). Kaempherol increases the levels of pro-apoptotic enzymes and proteins, such as cleaved caspase-9, -7, -3, p21, p53, Bax, PARP, and p-ATM [45,46] and decreased the levels of anti-apoptotic proteins Bcl2, polo-like kinase 1 (PLK-1), pAKT, phosphorylated insulin receptor substrate 1 (pIRS-1), phosphorylated mitogen-activated protein kinase (pMEK)1/2, cyclin-dependent kinase 1 (CDK1), cyclins A, B, D1, and E, and cathepsin D [10,40,41,45,46,47,48,49]. In triple-negative breast cancer cells (TNBC), kaempferol decrease cell migration and invasion stages when compared to non-TNBC cells (control) [42]. This is explained by the downregulation of RhoA and activation of Rac1 in TNBC cells, as well as through activation of human epidermal growth factor receptor-2 (HER2)-silence SK-BR-3 and ER/PR-silence in non-TNBC cells [42], which suggests that the antiproliferative action of kaempferol is triggered via the ER-dependent pathway that mediates cellular processes including development, differentiation, and proliferation [50]. In addition, kaempferol significantly activates mitogen-activated protein kinase (MAPK) cascades, which are key signaling pathways involved in the regulation of normal cell proliferation, survival, and differentiation. Indeed, kaempferol activates extracellular signal-regulated kinase (ERK), concomitantly with MEK1 and ELK1; while it reduces EMT and metastasis. The MAPK signaling pathway, when activated, leads to the transcription factor activator protein-1 (AP-1), cathepsin B and D, MMP-2 and -9 activation, that consequently reduces cell adhesion, migration, and invasion [51,52,53,54]. Also, kaempferol also lowers the glucose transporter 1 (GLUT1) mRNA levels and prevents the uptake of (3)*H*-deoxy-d-glucose ((3)H-DG) and monocarboxylate transporter 1 (MCT1)-mediated lactate cellular leading to extracellular lactate accumulation (Figure 2) [40].

### 4.2. Anti-Brain Cancer Activity

Glioblastoma is one of the most invasive and aggressive brain tumors, with a very poor prognosis, among other reasons, secondary to the development of resistance against current therapies [55]. It has been reported that Kaempferol inhibited both growth and migration of glioma cells, even when kaempferol was loaded to mucoadhesive nanoemulsion (KPF-MNE) or kaempferol-loaded nanoemulsion (KPF-NE) [55,56,57]. This flavonoid also triggers ROS generation and apoptosis, through reduction of the thioredoxin concentrations, superoxide dismutase activity, as well as to increase the levels of pro-inflammatory cytokines (interleukin-6, 8, chemokines, monocyte chemo-attractant protein-1), Bcl-2, cleaved caspase-3, -8, anti-apoptotic proteins survivin and XIAP, cleaved poly(ADP-ribose) polymerase expression, depolarization of mitochondrial membrane potential, and rapid reduction in phosphorylation of ERK and AKT [55,56,58].

### 4.3. Anti-Liver Cancer Activity

Hepatocellular carcinoma (HCC) is the most-encountered primary liver cancer among adults [59]. Kaempferol was revealed to significantly inhibit, in a dose-dependent manner, human hepatic cancer cells proliferation (HepG2, SK-HEP-1, Huh7). In addition, diethylnitrosamine and 2-acetylaminofluorene-induced HCC from rats treated with kaempferol combined to luteolin inhibited cell growth and induced cell death [60,61]. Indeed, kaempferol induces cell apoptosis and causes cell cycle arrest at the G2/M phase, therefore preventing cell migration and invasion. Kaempferol is also able to release cytochrome c via ROS generation triggering mitochondrial membrane potential loss and mitochondrial swelling and increasing the level of cleaved caspase-3 [59,60,61]. Kaempferol also decreases the expression level of miR-21, cytokine signaling 3 (SOCS3), signal transducer and activator of transcription 3 (STAT3), CDK1, cyclin B, PI3K/AKT/mTOR and p-mTOR signaling pathway, and hypoxia-inducible factor 1 (HIF-1) in human hepatic cancer cells and enhanced the expression of Janus kinase 1 (JAK1), tyrosine kinase 2 (Tyk2), STAT1/2, endogenous interferon (IFN)-α-regulated genes, phosphatase and tensin homologue (PTEN), microtubule-associated protein 1A/1B-light chain 3 (LC3-II), p44/42 MAPK, beclin 1, and autophagy-related gene (Atg) 5, 7, and 12 [60,61,62,63].

### 4.4. Anti-Colon Cancer Activity

Colorectal cancer is amongst the most frequently found cancers worldwide, with more than 1.8 million new cases per year [64]. Kaempferol was reported to possess cytotoxic effects on different human colorectal cancer cells lines, including HCT116, HT-29, HCT-15, LS174-R colon, and SW480 cells [64,65,66].

Even though 5-Fluorouracil is subjected to therapeutic failure due to resistance development, it is still the most recommended chemotherapeutic agent. Experimental studies combined kaempferol with 5-Fluorouracil in LS174-R cells and reported interesting antiproliferative effects [64]. In addition, kaempferol in combination with tumor necrosis factor ligand superfamily member (TRAIL) led to apoptosis in colon cancer cells, through up-regulation of TRAIL receptors and death receptor 5 (DR5) that improved the TRAIL activity [67]. Generally, kaempferol induces apoptosis and cell cycle arrest at G2/M, and reduces both cell migration and invasion [64,66]. Kaempferol also blocked ROS production and modulated the expression of MAPK, JAK/STAT3, PI3K/AKT, ATM, H2A histone family member X (H2AX), phospho-p38, p21, p53, PARP, caspase-3, -7, -8, -9, Bcl-2, p53 upregulated modulator of apoptosis (PUMA), the release of cytochrome c from mitochondria, connexin 43, ERK-1/2, and nuclear factor kappa B (NF-κB). Also, kaempferol significantly reduced insulin-like growth factor (IGF)-II secretion, and heregulin (HRG)-β, CDK2, CDK4, Cdc25C, Cdc2, cyclins B1, D1, E, A, and connexin 43 expressions. Finally, it also suppressed the phosphorylation of retinoblastoma protein and enhanced the PARP cleavages [64,65,66,68,69,70,71].

### 4.5. Anti-Prostate Cancer Activity

Prostate cancer is one of the leading causes of death among man and the need for more effective treatments has driven further research [72]. Kaempferol-3-*O*-rhamnoside dose-dependently inhibits prostate cancer cells proliferation [72], by upregulating the expression of caspase-8, -9, -3, and poly (ADP-ribose) polymerase proteins [72,73]. Granulocyte-macrophage colony-stimulating factor (GM-CSF) is known to activate the host immune system and to facilitate host immunosurveillance by the dendritic cells (DC), thereby representing a promising strategy to thwart prostate cancer [73]. Kaempferol has been shown to induce GM-CSF release in PC-3 cells that, in turn, increase the chemotaxis of DC through activation of phospholipase C (PLC), MEK1/2, and protein kinase C (PKC) [73]. Obviously, the transcriptome of prostate cancers cells is also markedly affected by kaempferol treatment as evidenced by the down-regulation of androgen receptor genes expression [74]. In rats, orally administered kaempferol showed no significant toxicity and significantly increased survival, in addition to reducing the growth of PCa xenografts in athymic nude mice [74].

### 4.6. Anti-Pancreatic Cancer Activity

Pancreatic cancer is amongst the most common cancer-related causes of deaths worldwide with the nastiest prognosis [75]. Kaempferol dose-dependently inhibits the growth of pancreatic cancer cells, SNU-213, Panic-1, and Miapaca-2, through inducing apoptosis [75] and effectively inhibiting cell migration, ERK1/2, epidermal growth factor receptor (EGFR)-related Src, and AKT pathways [76]. Kaempferol also improves the suppressive activity of regulatory T cells (Tregs) by increasing the FOXP3 expression level [77,78].

### 4.7. Anti-Blood Cancer Activity

Acute promyelocytic leukemia is a life-threatening blood cancer, characterized by a defect in cell growth and apoptotic pathways [79]. Kaempferol (12.5–100 μM) dose-dependently decreased cell viability in human leukemia cells, HL-60 and NB4 [79,80]. Kaempferol also promoted apoptosis, cell cycle arrest at the G2-M phase, and DNA damages [79,80,81,82,83,84], and down-regulated the expression of AKT, ABCB1, BCL2, and ABCC1 genes, protein expression associated with DNA repair system, as well as DNA-dependent serine/threonine protein kinase (DNA-PK), phosphate-ataxia-telangiectasia and Rad3-related (p-ATR), phosphate-ataxia-telangiectasia mutated (p-ATM), 14-3-3 proteins sigma (14-3-3σ), p53, MDC1, O(6)-methylguanine-DNA methyltransferase (MGMT), while up-regulating caspase-3, -8, p-p53, p-H2AX, and cytochrome c expression [79,80,81,82,85,86]. In a rat model of leukemia, kaempferol reduced the release of beta-hexosaminidase as a marker of degranulation in basophilic leukemia (RBL-2H3) cells [87], and increased the accumulation of mediators and the secretory granule development in human leukemic mast cells (HMC-1) [88].

### 4.8. Anti-Lung Cancer Activity

Lung cancer, such as non-small-cell lung cancer, displays a poor prognosis and is currently contributing to increasing the number of cancer-related deaths worldwide [89]. Kaempferol concentration dependently prevented the growth of lung adenocarcinoma A549 cells [90,91,92,93], decreased colony formation, and triggered apoptosis [94]. Kaempferol also markedly prevented cell migration, recovered the loss of E-cadherin, and suppressed EMT [89]. Kaempferol still downregulated the expression of claudin-2, AKT/PI3K phosphorylation, ERK pathways, Bcl-2, Bcl-xL, MEK1/2, MMP2, tissue inhibitor of metalloproteinases 2 (TIMP2), MAPK and up-regulated the expression of Bax, Fas, cleaved-caspase-3,-7,-8,-9, AIF (caspase-independent), and miR-340 transcription, involved in the apoptosis pathway [90,91,92,93,94,95]. In a lung metastasis model, kaempferol was also able to reduce the volume of subcutaneous xenograft and the number of metastasis compared to the control group [94]. In addition, it showed a significant effect in killing cancer cells by radiation in a BALB/c nude mouse xenograft model of A-549 cells [96].

### 4.9. Anti-Kidney Cancer Activity

Renal cell carcinoma (RCC) represents the most prevalent primary kidney cancer [97]. Kaempferol significantly inhibits cell growth and triggers apoptosis in RCC (786-O and 769-P cells) [98,99]. Kaempferol exerts its anticancer activity through preventing cell migration and invasion, inhibiting MMP-2 protein, downregulating AKT phosphorylation, and increasing the focal adhesion kinase (FAK) activity [97]). It also up-regulates cyclin B1 expression, PARP cleavages, and p21 expression and promotes activation of the EGFR/p38 signaling pathway [98,99].

### 4.10. Anti-Bladder Cancer Activity

Bladder cancer is becoming the most common type of cancer of the urinary tract [100]. Kaempferol can strongly and selectively inhibit bladder cancer cells by promoting cell cycle arrest and apoptosis [100,101,102,103]. Also, kaempferol acts by downregulating the PTEN/PI3K/AKT pathway, DNA methyltransferases (DNMT3B), CDK4, CyclinD1, Mcl-1, Bid, and Bcl-xL, and upregulating p53, p38, p21, p-ATM, p-BRCA1, DNA methylation, and Bid and Bax expression [100,102,103]. These in vitro findings were further validated by experiments in subcutaneous xenografted mouse models. Kaempferol significantly suppressed tumor growth as well as cancer metastasis and invasion in xenografted mice with regards to the untreated control compared to the control group mice, and caused downregulation of growth-related markers and c-Met/p38 signaling pathway, yet upregulated apoptosis markers [101].

### 4.11. Anti-Oral Cancer Activity

Oral squamous cell carcinomas (OSCC) is the sixth most prevalent cancer worldwide [104]. In in vitro studies, kaempferol displayed antiproliferative effect on pharynx (FaDu) and oral cavity carcinoma (PCI-13) [105], human esophageal squamous carcinoma (Eca-109), and human tongue squamous carcinoma (SCC4, SCC-1483, SCC-25, SCC-QLL1) cells, prevented clone formation and cell migration and invasion, and induced substantial apoptosis [104,106,107,108]. Kaempferol also caused cell cycle arrest at G0/G1 phase and downregulated Bcl-2, MMP-2, c-Jun, and hexokinase-2 expression. Kaempferol also increased glucose uptake, EGFR activation, ERK1/2 phosphorylation, and upregulated Bax, cleaved caspase-3, -9, and PARP [104,106,107,108]. Finally, the anticancer potency of kaempferol was further confirmed in a mice xenograft model, revealing the ability to significantly prevent the growth of tumor size coupled with a marked decrease in hexokinase-2 expression and EGFR activity in tumor tissues [107].

### 4.12. Anti-Bone Cancer Activity

Kaempferol dose-dependently inhibits the growth of human osteosarcoma cells U-2 OS, 143B, and HOB cells and the migration of human U-2 osteosarcoma (OS) cells with poor toxicity on hFOB cells, a human fetal osteoblast progenitor [109,110]. Kaempferol acts by downregulating the AP-1 DNA binding activity, MMP-2, -9, and urokinase plasminogen activator (uPA) that, in turn, reduces phosphorylated p38, ERK, and JNK [110]. In BALB/c(nu/nu) mice inoculated with human osteosarcoma cells (U-2 OS), kaempferol significantly decreased the number of viable cells and reduced the tumor size [109]. The in vivo anti-bone cancer effects of kaempferol have also been demonstrated in BALB/c(nu/nu) mice inoculated with U-2 OS cells [109].

### 4.13. Anti-Cervical Cancer Activity

Kaempferol was found to selectively prevent the growth of human cervical cancers cells, such as HeLa, multidrug-resistant human cervical carcinoma, KB-V1, and SiHa cells with regards to the normal cells and HFF cells [111,112,113,114]. Kaempferol also caused cell cycle arrest at the G2/M phase and apoptosis, correlated with downregulation of PI3K/AKT and human telomerase reverse transcriptase (hTERT) pathways, Pgp, Rh123 efflux, cyclin B1, NF-κB nuclear translocation, CDK1, Bcl-2, and upregulation of p53 with mitochondrial membrane potential disruption [111,112,113,114,115].

### 4.14. Anti-Stomach Cancer Activity

Experimental studies on stomach cancer revealed the antiproliferative activity of kaempferol on human gastric cancer cells (MKN28 and SGC7901) by promoting autophagy, cell cycle arrest at G2/M phase, and cell death [116,117]. The induced autophagic cell death was linked to the upregulation of Bax, cleaved caspase-3, -9, cleaved PARP, IRE1-JNK-CHOP signaling, and downregulation of p62, cyclin B1, Cdc25C, Bcl-2, CDK1, p-AKT, cyclooxygenase 2 (COX-2), and p-ERK expression [116,117].

### 4.15. Anti-Ovarian Cancer Activity

Experiments using human ovarian cancer cell lines (A2780/CP70, A2780/wt, SKOV-3, OVCAR-3) showed that kaempferol could inhibit tumor growth, proliferation, and angiogenesis by decreasing vascular endothelial growth factor (VEGF) expression [118]. Kaempferol also induces apoptosis and cell cycle arrest at G2/M phase via upregulation of Chk2/Cdc25C/Cdc2, DR5, DR4, JNK, CHOP, p38, p21, ERK1/2 proteins, caspase-3, -7, -8, Bad, Bax, and p53 proteins, with downregulation of hypoxia-inducible factor 1α (HIF-1α), a regulator of VEGF expression [118,119,120,121,122,123].

## 5. Conclusions

Cancer accounts among the most overbearing human health problems, relying on chemoprevention approaches as a way to diminish both incidence and mortality. The scrutiny of kaempferol extraordinary list of cancer-fighting properties highlights its full potential. These studies are promising, especially because kaempferol selectively inhibits cancerous cells without affecting healthy ones. In vitro studies unveiled the broad spectrum of kaempferol anticancer targets, including apoptosis, metastasis, inflammation, and angiogenesis. Therefore, cancer cells that often adapt to VEGF inhibition, following treatment with kaempferol, may not escape other detrimental actions induced by this natural flavonoid. Even though kaempferol is questionable as a cancer treatment, it seems to constitute an interesting option when it comes to safety. However, data on the long-term effect of kaempferol intake are scarce. Though kaempferol poor bioavailability represents a significant obstacle, the use of kaempferol-based nanoparticles has brought more hope on cancer chemoprevention strategies. Moreover, most of the research conducted on kaempferol anticancer potency was in vitro, making it difficult to draw a final conclusion on its usefulness. In vivo studies and clinical trials using an exact dose of kaempferol are scarce so far, thus stressing the need for more in-depth experiments varying the dose of kaempferol alone as well as using it with other flavonoids. These data will be of utmost interest to apprehend on kaempferol efficacy in the context of cancer.

## Figures and Tables

**Table 1:** Studies of anticancer activities of kaempferol in vitro.

Cancer Types	Mechanisms of Action	Cancer Cells Lines	Origin of Cells	References
Bladder	Downregulation: phosphorylated AKT (p-AKT), Cyclin D1, CDK4, Bid, Mcl-1 and Bcl-xL in human cells; DNMT3B expression in mouse cells Upregulation: p38, p53, p21, p-BRCA1, p-ATM, Bid, Bax expression in human cells; DNA methylation in mouse cells	SV-HUC-1 (human), T24 and 5637 (mouse)	Human, Mouse	[100,124]
Blood	Downregulation: p-ATM, phosphate-ataxia-telangiectasia, AKT, BCL2, ABCB1, and ABCC1 expression Upregulation: CASP3 and BAX/BCL-2 expression, subG1 population, Rad3-related (p-ATR), 14-3-3 proteins sigma (14-3-3σ), DNA-dependent serine, MDC1 protein, p53 and p-H2AX expression	HL-60, NB4	Human	[79,80]
Bone	Downregulation: migration, MMP-2, MMP-9, and uPA expression, ERK, p38, and JNK phosphorylation and DNA binding activity of AP-1, endoplasmic reticulum stress and mitochondrial signaling pathways	U-2 OS, HOB, 143B	Human	[109,110]
Brain	Apoptosis Downregulation: phosphorylation of ERK, AKT, anti-apoptotic proteins XIAP and survivin expression, depolarization of mitochondrial membrane potential Upregulation: caspase-3 activity	C6, A172	Rats, Human	[55,57]
Breast	Downregulation: Bcl2, E2, EMT-markers (N-cadherin, E-cadherin, Slug, and Snail), cathepsin D, cyclin D1, cyclin E, pAkt, pMEK1/2, pIRS-1, RhoA and Rac1 activation of ER/PR-silence and HER2-silence SK-BR-3 Upregulation: p21, bax γH2AX, cleaved caspase-3&-9, and p-ATM Suppression of migration and invasion Apoptosis, cell cycle arrest at G2/M and DNA damage, reduced cell migration and invasion ability	Triple-negative BC (TNBC) cell MDA-MB-231, MCF-7	Human	[10,41,42,47,51,60]
Cervical	Downregulation: PI3K/AKT and hTERT pathways Upregulation: mitochondrial membrane potential disruption, intracellular free calcium elevation Apoptosis	HeLa, SiHa	Human	[111,112,114]
Colon	Downregulation: CDK2, CDK4, cyclins D1, cyclin B1, cyclin E, cyclin A, Cdc25C, Cdc2, IGF-I&-II secretion, heregulin (HRG)-β expression and HRG-β-induced phosphorylation of the AKT, ERK-1/2, IGF-IR, and ErbB3 Upregulation: caspase-3,-8,-9, p21, p53, phospho-p38 MAPK and enhanced the PARP cleavages, JAK/STAT3, MAPK, PI3K/AKT, and NF-κB expression Blocked ROS generation, cell cycle arrest at G1 and G2/M arrest, and cell migration	LS174, HCT15, HCT116, SW480, HT-29	Human	[64,65,66,125]
Kidney	Downregulation: MMP-2, AKT phosphorylation and FAK	786-O	Human	[97]
Liver	Downregulation: mitochondrial membrane potential, mitochondrial swelling, SOCS3, STAT3, miR-21, PI3K/AKT/mTOR signaling pathway Upregulation: PTEN, caspase-3, JAK1, Tyk2, STAT1/2, endogenous IFN-α-regulated genes expression	Hepatoma HepG2	Rat, Human	[59,60,63]
Lung	Downregulation: AKT/PI3K and ERK pathways, TIMP2, and MMP2 phosphorylation, Bcl-2, cyclin D1, claudin-2 expression Inhibited STAT3 factor binding Upregulation: PTEN, Bax, miR-340, Fas, cleaved-caspases 3, 8, and 9, and cleaved-PARP Apoptosis, cell cycle arrest at G2/M, prevent migration and invasion	A549, HCCC9810, QBC939	Human, mice	[90,92,94,96]
Oral	Suppress migration and invasion Downregulation: MMP-2, TIMP-2 mRNA, c-Jun activity, ERK1/2 phosphorylation	SCC4	Human	[104]
Ovarian	Upregulation: DR4, DR5, p53, p38, ERK1/2, CHOP, JNK, death receptors/FADD/Caspase-8 pathway Downregulation: anti-apoptotic proteins	A2780/CP70, OVCAR-3, SKOV-3	Human	[119,120,121]
Pancreatic	Downregulation: EGFR-related AKT, Src, and ERK1/2, pathways Upregulation: suppressive function of regulatory T cells (Tregs), FOXP3 expression Block cell migration	Miapaca-2, Panc-1, SNU-213, Treg cells	Human, Rats	[76,77]
Prostate	Downregulation: androgen receptor expression Upregulation: caspase-8, -9, -3 and poly (ADP-ribose) polymerase proteins cleavage	C4-2, LNCaP	Mice, Human	[72,74]
Stomach	Induce significant apoptosis and cell cycle arrest at G2/M Downregulation: COX-2, Bcl-2 p-ERK, p-AKT expression Upregulation: Bax, cleaved caspase-3 and -9	MKN28 and SGC7901	Human	[116]

## References

[1] Li H., Ji H.-S., Kang J.-H., Shin D.-H., Park H.-Y., Choi M.-S., Lee C.-H., Lee I.-K., Yun B.-S., Jeong T.-S. (2015). Soy Leaf Extract Containing Kaempferol Glycosides and Pheophorbides Improves Glucose Homeostasis by Enhancing Pancreatic β-Cell Function and Suppressing Hepatic Lipid Accumulation in db/db Mice. J. Agric. Food. Chem..

[2] Bhagwat S., Haytowitz D.B., Holden J.M., Nutrient Data Laboratory, B.H.N.R.C., ARS, USDA (2014). USDA Database for the Flavonoid Content of Selected Foods, Release 3.1. USDA Special Interest Databases on Flavonoids.

[3] Rajendran P., Rengarajan T., Nandakumar N., Palaniswami R., Nishigaki Y., Nishigaki I. (2014). Kaempferol, a potential cytostatic and cure for inflammatory disorders. Eur. J. Med. Chem..

[4] Sharifi-Rad M., Fokou P.V.T., Sharopov F., Martorell M., Ademiluyi A.O., Rajkovic J., Salehi B., Martins N., Iriti M., Sharifi-Rad J. (2018). Antiulcer agents: From plant extracts to phytochemicals in healing promotion. Molecules.

[5] Calderon-Montano J.M., Burgos-Moron E., Perez-Guerrero C., Lopez-Lazaro M. (2011). A review on the dietary flavonoid kaempferol. Mini Rev. Med. Chem..

[6] Pei J., Chen A., Zhao L., Cao F., Ding G., Xiao W. (2017). One-Pot Synthesis of Hyperoside by a Three-Enzyme Cascade Using a UDP-Galactose Regeneration System. J. Agric. Food. Chem..

[7] Neuhouser M.L. (2004). Dietary flavonoids and cancer risk: Evidence from human population studies. Nutr. Cancer.

[8] Weng C.J., Yen G.C. (2012). Flavonoids, a ubiquitous dietary phenolic subclass, exert extensive in vitro anti-invasive and in vivo anti-metastatic activities. Cancer Metastasis Rev..

[9] Elsharkawy E.R. (2017). Isolation of phytoconstituents and evaluation of anticancer and antioxidant potential of launaea mucronata (forssk.) muschl. subsp. Pak. J. Pharm. Sci..

[10] Yi X., Zuo J., Tan C., Xian S., Luo C., Chen S., Yu L., Luo Y. (2016). Kaempferol, a flavonoid compound from gynura medica induced apoptosis and growth inhibition in mcf-7 breast cancer cell. Afr. J. Tradit. AJTCAM.

[11] Mishra A.P., Salehi B., Sharifi-Rad M., Pezzani R., Kobarfard F., Sharifi-Rad J., Nigam M. (2018). Programmed Cell Death, from a Cancer Perspective: An Overview. Mol. Diagn. Ther..

[12] Imran M., Rauf A., Shah Z.A., Saeed F., Imran A., Arshad M.U., Ahmad B., Bawazeer S., Atif M., Peters D.G. (2019). Chemo-preventive and therapeutic effect of the dietary flavonoid kaempferol: A comprehensive review. Phytother. Res..

[13] Marfe G., Tafani M., Indelicato M., Sinibaldi-Salimei P., Reali V., Pucci B., Fini M., Russo M.A. (2009). Kaempferol induces apoptosis in two different cell lines via Akt inactivation, Bax and SIRT3 activation, and mitochondrial dysfunction. J. Cell. Biochem..

[14] Kim K.Y., Jang W.Y., Lee J.Y., Jun D.Y., Ko J.Y., Yun Y.H., Kim Y.H. (2016). Kaempferol Activates G(2)-Checkpoint of the Cell Cycle Resulting in G(2)-Arrest and Mitochondria-Dependent Apoptosis in Human Acute Leukemia Jurkat T Cells. J. Microbiol. Biotechnol..

[15] Kim B., Jung J.W., Jung J., Han Y., Suh D.H., Kim H.S., Dhanasekaran D.N., Song Y.S. (2017). PGC1alpha induced by reactive oxygen species contributes to chemoresistance of ovarian cancer cells. Oncotarget.

[16] Lehtonen H.-M., Lehtinen O., Suomela J.-P., Viitanen M., Kallio H. (2009). Flavonol glycosides of sea buckthorn (Hippophae rhamnoides ssp. sinensis) and lingonberry (Vaccinium vitis-idaea) are bioavailable in humans and monoglucuronidated for excretion. J. Agric. Food. Chem..

[17] Crespy V., Morand C., Besson C., Cotelle N., Vezin H., Demigne C., Remesy C. (2003). The splanchnic metabolism of flavonoids highly differed according to the nature of the compound. Am. J. Physiol..

[18] Cao J., Zhang Y., Chen W., Zhao X. (2010). The relationship between fasting plasma concentrations of selected flavonoids and their ordinary dietary intake. Br. J. Nutr..

[19] Yodogawa S., Arakawa T., Sugihara N., Furuno K. (2003). Glucurono- and sulfo-conjugation of kaempferol in rat liver subcellular preparations and cultured hepatocytes. Biol. Pharm. Bull..

[20] Barve A., Chen C., Hebbar V., Desiderio J., Saw C.L., Kong A.N. (2009). Metabolism, oral bioavailability and pharmacokinetics of chemopreventive kaempferol in rats. Biopharm. Drug Dispos..

[21] Bonetti A., Marotti I., Dinelli G. (2007). Urinary excretion of kaempferol from common beans (*Phaseolus vulgaris* L.) in humans. Int. J. Food Sci. Nutr..

[22] DuPont M.S., Day A.J., Bennett R.N., Mellon F.A., Kroon P.A. (2004). Absorption of kaempferol from endive, a source of kaempferol-3-glucuronide, in humans. Eur. J. Clin. Nutr..

[23] Wang F.M., Yao T.W., Zeng S. (2003). Disposition of quercetin and kaempferol in human following an oral administration of Ginkgo biloba extract tablets. Eur. J. Drug Metab. Pharmacokinet..

[24] Hein E.M., Rose K., van’t Slot G., Friedrich A.W., Humpf H.U. (2008). Deconjugation and degradation of flavonol glycosides by pig cecal microbiota characterized by Fluorescence in situ hybridization (FISH). J. Agric. Food Chem..

[25] Labib S., Hummel S., Richling E., Humpf H.U., Schreier P. (2006). Use of the pig caecum model to mimic the human intestinal metabolism of hispidulin and related compounds. Mol. Nutr. Food Res..

[26] Kampkotter A., Gombitang Nkwonkam C., Zurawski R.F., Timpel C., Chovolou Y., Watjen W., Kahl R. (2007). Effects of the flavonoids kaempferol and fisetin on thermotolerance, oxidative stress and FoxO transcription factor DAF-16 in the model organism Caenorhabditis elegans. Arch. Toxicol..

[27] Verma A.R., Vijayakumar M., Mathela C.S., Rao C.V. (2009). In vitro and in vivo antioxidant properties of different fractions of *Moringa oleifera* leaves. Food Chem. Toxicol..

[28] López-Lázaro M. (2010). A new view of carcinogenesis and an alternative approach to cancer therapy. Mol. Med..

[29] Salehi B., Martorell M., Arbiser J.L., Sureda A., Martins N., Maurya P.K., Sharifi-Rad M., Kumar P., Sharifi-Rad J. (2018). Antioxidants: Positive or Negative Actors?. Biomolecules.

[30] Sharifi-Rad J., Sharifi-Rad M., Salehi B., Iriti M., Roointan A., Mnayer D., Soltani-Nejad A., Afshari A. (2018). In vitro and in vivo assessment of free radical scavenging and antioxidant activities of *Veronica persica* Poir. Cell. Mol. Biol..

[31] Salehi B., Valussi M., Jugran A.K., Martorell M., Ramírez-Alarcón K., Stojanović-Radić Z.Z., Antolak H., Kręgiel D., Mileski K.S., Sharifi-Rad M. (2018). *Nepeta* species: From farm to food applications and phytotherapy. Trends Food Sci. Technol..

[32] Wang L., Tu Y.C., Lian T.W., Hung J.T., Yen J.H., Wu M.J. (2006). Distinctive antioxidant and antiinflammatory effects of flavonols. J. Agric. Food Chem..

[33] Heijnen C.G., Haenen G.R., van Acker F.A., van der Vijgh W.J., Bast A. (2001). Flavonoids as peroxynitrite scavengers: The role of the hydroxyl groups. Toxicol. In Vitro.

[34] Klaunig J.E., Kamendulis L.M. (2004). The role of oxidative stress in carcinogenesis. Annu. Rev. Pharmacol. Toxicol..

[35] Ozyurek M., Bektasoglu B., Guclu K., Apak R. (2009). Measurement of xanthine oxidase inhibition activity of phenolics and flavonoids with a modified cupric reducing antioxidant capacity (CUPRAC) method. Anal. Chim. Acta.

[36] Doronicheva N., Yasui H., Sakurai H. (2007). Chemical structure-dependent differential effects of flavonoids on the catalase activity as evaluated by a chemiluminescent method. Biol. Pharm. Bull..

[37] Hong J.T., Yen J.H., Wang L., Lo Y.H., Chen Z.T., Wu M.J. (2009). Regulation of heme oxygenase-1 expression and MAPK pathways in response to kaempferol and rhamnocitrin in PC12 cells. Toxicol. Appl. Pharmacol..

[38] Mira L., Fernandez M.T., Santos M., Rocha R., Florencio M.H., Jennings K.R. (2002). Interactions of flavonoids with iron and copper ions: A mechanism for their antioxidant activity. Free Radic. Res..

[39] Ren J., Meng S., Lekka Ch E., Kaxiras E. (2008). Complexation of flavonoids with iron: Structure and optical signatures. J. Phys. Chem. B.

[40] Azevedo C., Correia-Branco A., Araujo J.R., Guimaraes J.T., Keating E., Martel F. (2015). The chemopreventive effect of the dietary compound kaempferol on the MCF-7 human breast cancer cell line is dependent on inhibition of glucose cellular uptake. Nutr. Cancer.

[41] Zhu L., Xue L. (2018). Kaempferol suppresses proliferation and induces cell cycle arrest, apoptosis, and DNA damage in breast cancer cells. Oncol. Res..

[42] Li S., Yan T., Deng R., Jiang X., Xiong H., Wang Y., Yu Q., Wang X., Chen C., Zhu Y. (2017). Low dose of kaempferol suppresses the migration and invasion of triple-negative breast cancer cells by downregulating the activities of RhoA and Rac1. OncoTargets Ther..

[43] Lee S.B., Shin J.S., Han H.S., Lee H.H., Park J.C., Lee K.T. (2018). Kaempferol 7-O-beta-D-glucoside isolated from the leaves of Cudrania tricuspidata inhibits LPS-induced expression of pro-inflammatory mediators through inactivation of NF-kappaB, AP-1, and JAK-STAT in RAW 264.7 macrophages. Chem. Biol. Interact..

[44] Lee G.A., Choi K.C., Hwang K.A. (2018). Treatment with Phytoestrogens Reversed Triclosan and Bisphenol A-Induced Anti-Apoptosis in Breast Cancer Cells. Biomol. Ther..

[45] Diantini A., Subarnas A., Lestari K., Halimah E., Susilawati Y., Supriyatna S., Julaeha E., Achmad T.H., Suradji E.W., Yamazaki C. (2012). Kaempferol-3-O-rhamnoside isolated from the leaves of Schima wallichii Korth. inhibits MCF-7 breast cancer cell proliferation through activation of the caspase cascade pathway. Oncol. Lett..

[46] Tsiklauri L., An G., Ruszaj D.M., Alaniya M., Kemertelidze E., Morris M.E. (2011). Simultaneous determination of the flavonoids robinin and kaempferol in human breast cancer cells by liquid chromatography-tandem mass spectrometry. J. Pharm. Biomed. Anal..

[47] Kim S.H., Hwang K.A., Choi K.C. (2016). Treatment with kaempferol suppresses breast cancer cell growth caused by estrogen and triclosan in cellular and xenograft breast cancer models. J. Nutr. Biochem..

[48] Kang G.Y., Lee E.R., Kim J.H., Jung J.W., Lim J., Kim S.K., Cho S.G., Kim K.P. (2009). Downregulation of PLK-1 expression in kaempferol-induced apoptosis of MCF-7 cells. Eur. J. Pharmacol..

[49] Choi E.J., Ahn W.S. (2008). Kaempferol induced the apoptosis via cell cycle arrest in human breast cancer MDA-MB-453 cells. Nutr. Res. Pract..

[50] Oh S.M., Kim Y.P., Chung K.H. (2006). Biphasic effects of kaempferol on the estrogenicity in human breast cancer cells. Arch. Pharmacal Res..

[51] Lee G.A., Choi K.C., Hwang K.A. (2017). Kaempferol, a phytoestrogen, suppressed triclosan-induced epithelial-mesenchymal transition and metastatic-related behaviors of MCF-7 breast cancer cells. Environ. Toxicol. Pharmacol..

[52] Zheng L., Zhu L., Zhao M., Shi J., Li Y., Yu J., Jiang H., Wu J., Tong Y., Liu Y. (2016). In Vivo Exposure of Kaempferol Is Driven by Phase II Metabolic Enzymes and Efflux Transporters. AAPS.

[53] Li C., Zhao Y., Yang D., Yu Y., Guo H., Zhao Z., Zhang B., Yin X. (2015). Inhibitory effects of kaempferol on the invasion of human breast carcinoma cells by downregulating the expression and activity of matrix metalloproteinase-9. Biochem. Cell Biol..

[54] Kim B.W., Lee E.R., Min H.M., Jeong H.S., Ahn J.Y., Kim J.H., Choi H.Y., Choi H., Kim E.Y., Park S.P. (2008). Sustained ERK activation is involved in the kaempferol-induced apoptosis of breast cancer cells and is more evident under 3-D culture condition. Cancer Boil. Ther..

[55] Jeong J.C., Kim M.S., Kim T.H., Kim Y.K. (2009). Kaempferol induces cell death through ERK and Akt-dependent down-regulation of XIAP and survivin in human glioma cells. Neurochem. Res..

[56] Sharma V., Joseph C., Ghosh S., Agarwal A., Mishra M.K., Sen E. (2007). Kaempferol induces apoptosis in glioblastoma cells through oxidative stress. Mol. Cancer Ther..

[57] Colombo M., Figueiro F., de Fraga Dias A., Teixeira H.F., Battastini A.M.O., Koester L.S. (2018). Kaempferol-loaded mucoadhesive nanoemulsion for intranasal administration reduces glioma growth in vitro. Int. J. Pharm..

[58] Siegelin M.D., Reuss D.E., Habel A., Herold-Mende C., von Deimling A. (2008). The flavonoid kaempferol sensitizes human glioma cells to TRAIL-mediated apoptosis by proteasomal degradation of survivin. Mol. Cancer Ther..

[59] Seydi E., Salimi A., Rasekh H.R., Mohsenifar Z., Pourahmad J. (2018). Selective Cytotoxicity of Luteolin and Kaempferol on Cancerous Hepatocytes Obtained from Rat Model of Hepatocellular Carcinoma: Involvement of ROS-Mediated Mitochondrial Targeting. Nutr. Cancer.

[60] Zhu G., Liu X., Li H., Yan Y., Hong X., Lin Z. (2018). Kaempferol inhibits proliferation, migration, and invasion of liver cancer HepG2 cells by down-regulation of microRNA-21. Int. J. Immunopathol. Pharmacol..

[61] Mylonis I., Lakka A., Tsakalof A., Simos G. (2010). The dietary flavonoid kaempferol effectively inhibits HIF-1 activity and hepatoma cancer cell viability under hypoxic conditions. Biochem. Biophys. Res. Commun..

[62] Huang W.W., Tsai S.C., Peng S.F., Lin M.W., Chiang J.H., Chiu Y.J., Fushiya S., Tseng M.T., Yang J.S. (2013). Kaempferol induces autophagy through AMPK and AKT signaling molecules and causes G2/M arrest via downregulation of CDK1/cyclin B in SK-HEP-1 human hepatic cancer cells. Int. J. Oncol..

[63] Wonganan O., He Y.J., Shen X.F., Wongkrajang K., Suksamrarn A., Zhang G.L., Wang F. (2017). 6-Hydroxy-3-O-methyl-kaempferol 6-O-glucopyranoside potentiates the anti-proliferative effect of interferon alpha/beta by promoting activation of the JAK/STAT signaling by inhibiting SOCS3 in hepatocellular carcinoma cells. Toxicol. Appl. Pharmacol..

[64] Riahi-Chebbi I., Souid S., Othman H., Haoues M., Karoui H., Morel A., Srairi-Abid N., Essafi M., Essafi-Benkhadir K. (2019). The Phenolic compound Kaempferol overcomes 5-fluorouracil resistance in human resistant LS174 colon cancer cells. Sci. Rep..

[65] Choi J.B., Kim J.H., Lee H., Pak J.N., Shim B.S., Kim S.H. (2018). Reactive Oxygen Species and p53 Mediated Activation of p38 and Caspases is Critically Involved in Kaempferol Induced Apoptosis in Colorectal Cancer Cells. J. Agric. Food Chem..

[66] Lee H.S., Cho H.J., Yu R., Lee K.W., Chun H.S., Park J.H. (2014). Mechanisms underlying apoptosis-inducing effects of Kaempferol in HT-29 human colon cancer cells. Int. J. Mol. Sci..

[67] Yoshida T., Konishi M., Horinaka M., Yasuda T., Goda A.E., Taniguchi H., Yano K., Wakada M., Sakai T. (2008). Kaempferol sensitizes colon cancer cells to TRAIL-induced apoptosis. Biochem. Biophys. Res. Commun..

[68] Deepa M., Sureshkumar T., Satheeshkumar P.K., Priya S. (2013). Antioxidant rich Morus alba leaf extract induces apoptosis in human colon and breast cancer cells by the downregulation of nitric oxide produced by inducible nitric oxide synthase. Nutr. Cancer.

[69] Nirmala P., Ramanathan M. (2011). Effect of kaempferol on lipid peroxidation and antioxidant status in 1,2-dimethyl hydrazine induced colorectal carcinoma in rats. Eur. J. Pharmacol..

[70] Li W., Du B., Wang T., Wang S., Zhang J. (2009). Kaempferol induces apoptosis in human HCT116 colon cancer cells via the Ataxia-Telangiectasia Mutated-p53 pathway with the involvement of p53 Upregulated Modulator of Apoptosis. Chem. Biol. Interact..

[71] Nakamura Y., Chang C.C., Mori T., Sato K., Ohtsuki K., Upham B.L., Trosko J.E. (2005). Augmentation of differentiation and gap junction function by kaempferol in partially differentiated colon cancer cells. Carcinogenesis.

[72] Halimah E., Diantini A., Destiani D.P., Pradipta I.S., Sastramihardja H.S., Lestari K., Subarnas A., Abdulah R., Koyama H. (2015). Induction of caspase cascade pathway by kaempferol-3-O-rhamnoside in LNCaP prostate cancer cell lines. Biomed. Rep..

[73] Bandyopadhyay S., Romero J.R., Chattopadhyay N. (2008). Kaempferol and quercetin stimulate granulocyte-macrophage colony-stimulating factor secretion in human prostate cancer cells. Mol. Cell. Endocrinol..

[74] Mamouni K., Zhang S., Li X., Chen Y., Yang Y., Kim J., Bartlett M.G., Coleman I.M., Nelson P.S., Kucuk O. (2018). A novel flavonoid composition targets androgen receptor signaling and inhibits prostate cancer growth in preclinical models. Neoplasia.

[75] Zhang Y., Chen A.Y., Li M., Chen C., Yao Q. (2008). Ginkgo biloba extract kaempferol inhibits cell proliferation and induces apoptosis in pancreatic cancer cells. J. Surg. Res..

[76] Lee J., Kim J.H. (2016). Kaempferol Inhibits Pancreatic Cancer Cell Growth and Migration through the Blockade of EGFR-Related Pathway In Vitro. PLoS ONE.

[77] Lin F., Luo X., Tsun A., Li Z., Li D., Li B. (2015). Kaempferol enhances the suppressive function of Treg cells by inhibiting FOXP3 phosphorylation. Int. Immunopharmacol..

[78] Nothlings U., Murphy S.P., Wilkens L.R., Boeing H., Schulze M.B., Bueno-de-Mesquita H.B., Michaud D.S., Roddam A., Rohrmann S., Tjonneland A. (2008). A food pattern that is predictive of flavonol intake and risk of pancreatic cancer. Am. J. Clin. Nutr..

[79] Moradzadeh M., Tabarraei A., Sadeghnia H.R., Ghorbani A., Mohamadkhani A., Erfanian S., Sahebkar A. (2018). Kaempferol increases apoptosis in human acute promyelocytic leukemia cells and inhibits multidrug resistance genes. J. Cell. Biochem..

[80] Wu L.Y., Lu H.F., Chou Y.C., Shih Y.L., Bau D.T., Chen J.C., Hsu S.C., Chung J.G. (2015). Kaempferol induces DNA damage and inhibits DNA repair associated protein expressions in human promyelocytic leukemia HL-60 cells. Am. J. Chin. Med..

[81] Bestwick C.S., Milne L., Duthie S.J. (2007). Kaempferol induced inhibition of HL-60 cell growth results from a heterogeneous response, dominated by cell cycle alterations. Chem. Biol. Interact..

[82] Bestwick C.S., Milne L., Pirie L., Duthie S.J. (2005). The effect of short-term kaempferol exposure on reactive oxygen levels and integrity of human (HL-60) leukaemic cells. BBA.

[83] Rusak G., Gutzeit H.O., Müller J.L. (2005). Structurally related flavonoids with antioxidative properties differentially affect cell cycle progression and apoptosis of human acute leukemia cells. Nutr. Res..

[84] Casagrande F., Darbon J.M. (2001). Effects of structurally related flavonoids on cell cycle progression of human melanoma cells: Regulation of cyclin-dependent kinases CDK2 and CDK1. Biochem. Pharmacol..

[85] Benyahia S., Benayache S., Benayache F., Quintana J., Lopez M., Leon F., Hernandez J.C., Estevez F., Bermejo J. (2004). Isolation from Eucalyptus occidentalis and identification of a new kaempferol derivative that induces apoptosis in human myeloid leukemia cells. J. Nat. Prod..

[86] Chen D., Daniel K.G., Chen M.S., Kuhn D.J., Landis-Piwowar K.R., Dou Q.P. (2005). Dietary flavonoids as proteasome inhibitors and apoptosis inducers in human leukemia cells. Biochem. Pharmacol..

[87] Xu F., Matsuda H., Hata H., Sugawara K., Nakamura S., Yoshikawa M. (2009). Structures of new flavonoids and benzofuran-type stilbene and degranulation inhibitors of rat basophilic leukemia cells from the Brazilian herbal medicine Cissus sicyoides. Chem. Pharm. Bull..

[88] Alexandrakis M., Letourneau R., Kempuraj D., Kandere-Grzybowska K., Huang M., Christodoulou S., Boucher W., Seretakis D., Theoharides T.C. (2003). Flavones inhibit proliferation and increase mediator content in human leukemic mast cells (HMC-1). Eur. J. Haematol..

[89] Jo E., Park S.J., Choi Y.S., Jeon W.K., Kim B.C. (2015). Kaempferol Suppresses Transforming Growth Factor-beta1-Induced Epithelial-to-Mesenchymal Transition and Migration of A549 Lung Cancer Cells by Inhibiting Akt1-Mediated Phosphorylation of Smad3 at Threonine-179. Neoplasia.

[90] Sonoki H., Tanimae A., Endo S., Matsunaga T., Furuta T., Ichihara K., Ikari A. (2017). Kaempherol and Luteolin Decrease Claudin-2 Expression Mediated by Inhibition of STAT3 in Lung Adenocarcinoma A549 Cells. Nutrients.

[91] Boadi W.Y., Lo A. (2018). Effects of Quercetin, Kaempferol, and Exogenous Glutathione on Phospho- and Total-AKT in 3T3-L1 Preadipocytes. J. Diet. Suppl..

[92] Han X., Liu C.F., Gao N., Zhao J., Xu J. (2018). Kaempferol suppresses proliferation but increases apoptosis and autophagy by up-regulating microRNA-340 in human lung cancer cells. Biomed. Pharmacother..

[93] Nguyen T.T., Tran E., Ong C.K., Lee S.K., Do P.T., Huynh T.T., Nguyen T.H., Lee J.J., Tan Y., Ong C.S. (2003). Kaempferol-induced growth inhibition and apoptosis in A549 lung cancer cells is mediated by activation of MEK-MAPK. J. Cell. Physiol..

[94] Qin Y., Cui W., Yang X., Tong B. (2016). Kaempferol inhibits the growth and metastasis of cholangiocarcinoma in vitro and in vivo. Acta Biochim. Biophy. Sin..

[95] Leung H.W., Lin C.J., Hour M.J., Yang W.H., Wang M.Y., Lee H.Z. (2007). Kaempferol induces apoptosis in human lung non-small carcinoma cells accompanied by an induction of antioxidant enzymes. Food Chem. Toxicol..

[96] Kuo W.T., Tsai Y.C., Wu H.C., Ho Y.J., Chen Y.S., Yao C.H., Yao C.H. (2015). Radiosensitization of non-small cell lung cancer by kaempferol. Oncol. Rep..

[97] Hung T.-W., Chen P.-N., Wu H.-C., Wu S.-W., Tsai P.-Y., Hsieh Y.-S., Chang H.-R. (2017). Kaempferol Inhibits the Invasion and Migration of Renal Cancer Cells through the Downregulation of AKT and FAK Pathways. Int. J. Med. Sci..

[98] An G., Gallegos J., Morris M.E. (2011). The bioflavonoid kaempferol is an Abcg2 substrate and inhibits Abcg2-mediated quercetin efflux. Drug Metab. Dispos..

[99] Song W., Dang Q., Xu D., Chen Y., Zhu G., Wu K., Zeng J., Long Q., Wang X., He D. (2014). Kaempferol induces cell cycle arrest and apoptosis in renal cell carcinoma through EGFR/p38 signaling. Oncol. Rep..

[100] Wu P., Meng X., Zheng H., Zeng Q., Chen T., Wang W., Zhang X., Su J. (2018). Kaempferol Attenuates ROS-Induced Hemolysis and the Molecular Mechanism of Its Induction of Apoptosis on Bladder Cancer. Molecules.

[101] Dang Q., Song W., Xu D., Ma Y., Li F., Zeng J., Zhu G., Wang X., Chang L.S., He D. (2015). Kaempferol suppresses bladder cancer tumor growth by inhibiting cell proliferation and inducing apoptosis. Mol. Carcinog..

[102] Garcia R., Gonzalez C.A., Agudo A., Riboli E. (1999). High intake of specific carotenoids and flavonoids does not reduce the risk of bladder cancer. Nutr. Cancer.

[103] Xie F., Su M., Qiu W., Zhang M., Guo Z., Su B., Liu J., Li X., Zhou L. (2013). Kaempferol promotes apoptosis in human bladder cancer cells by inducing the tumor suppressor, PTEN. Int. J. Mol. Sci..

[104] Lin C.W., Chen P.N., Chen M.K., Yang W.E., Tang C.H., Yang S.F., Hsieh Y.S. (2013). Kaempferol reduces matrix metalloproteinase-2 expression by down-regulating ERK1/2 and the activator protein-1 signaling pathways in oral cancer cells. PLoS ONE.

[105] Swanson H.I., Choi E.Y., Helton W.B., Gairola C.G., Valentino J. (2014). Impact of apigenin and kaempferol on human head and neck squamous cell carcinoma. Oral Surg. Oral Med. Oral Pathol. Oral Radiol..

[106] Li R.J., Mei J.Z., Liu G.J. (2011). [Kaempferol-induced apoptosis of human esophageal squamous carcinoma Eca-109 cells and the mechanism]. J. South Med. Univ..

[107] Yao S., Wang X., Li C., Zhao T., Jin H., Fang W. (2016). Kaempferol inhibits cell proliferation and glycolysis in esophagus squamous cell carcinoma via targeting EGFR signaling pathway. Tumour Boil..

[108] Kang J.W., Kim J.H., Song K., Kim S.H., Yoon J.H., Kim K.S. (2010). Kaempferol and quercetin, components of Ginkgo biloba extract (EGb 761), induce caspase-3-dependent apoptosis in oral cavity cancer cells. Phytother. Res..

[109] Huang W.W., Chiu Y.J., Fan M.J., Lu H.F., Yeh H.F., Li K.H., Chen P.Y., Chung J.G., Yang J.S. (2010). Kaempferol induced apoptosis via endoplasmic reticulum stress and mitochondria-dependent pathway in human osteosarcoma U-2 OS cells. Mol. Nutr. Food Res..

[110] Chen H.J., Lin C.M., Lee C.Y., Shih N.C., Peng S.F., Tsuzuki M., Amagaya S., Huang W.W., Yang J.S. (2013). Kaempferol suppresses cell metastasis via inhibition of the ERK-p38-JNK and AP-1 signaling pathways in U-2 OS human osteosarcoma cells. Oncol. Rep..

[111] Kashafi E., Moradzadeh M., Mohamadkhani A., Erfanian S. (2017). Kaempferol increases apoptosis in human cervical cancer HeLa cells via PI3K/AKT and telomerase pathways. Biomed. Pharmacother..

[112] Liao W., Chen L., Ma X., Jiao R., Li X., Wang Y. (2016). Protective effects of kaempferol against reactive oxygen species-induced hemolysis and its antiproliferative activity on human cancer cells. Eur. J. Med. Chem..

[113] Limtrakul P., Khantamat O., Pintha K. (2005). Inhibition of P-glycoprotein function and expression by kaempferol and quercetin. J. Chemother..

[114] Tu L.Y., Bai H.H., Cai J.Y., Deng S.P. (2016). The mechanism of kaempferol induced apoptosis and inhibited proliferation in human cervical cancer SiHa cell: From macro to nano. Scanning.

[115] Xu W., Liu J., Li C., Wu H.Z., Liu Y.W. (2008). Kaempferol-7-O-beta-D-glucoside (KG) isolated from *Smilax china* L. rhizome induces G2/M phase arrest and apoptosis on HeLa cells in a p53-independent manner. Cancer Lett..

[116] Song H., Bao J., Wei Y., Chen Y., Mao X., Li J., Yang Z., Xue Y. (2015). Kaempferol inhibits gastric cancer tumor growth: An in vitro and in vivo study. Oncol. Rep..

[117] Kim T.W., Lee S.Y., Kim M., Cheon C., Ko S.G. (2018). Kaempferol induces autophagic cell death via IRE1-JNK-CHOP pathway and inhibition of G9a in gastric cancer cells. Cell Death Dis..

[118] Luo H., Rankin G.O., Liu L., Daddysman M.K., Jiang B.H., Chen Y.C. (2009). Kaempferol inhibits angiogenesis and VEGF expression through both HIF dependent and independent pathways in human ovarian cancer cells. Nutr. Cancer.

[119] Gao Y., Yin J., Rankin G.O., Chen Y.C. (2018). Kaempferol Induces G2/M Cell Cycle Arrest via Checkpoint Kinase 2 and Promotes Apoptosis via Death Receptors in Human Ovarian Carcinoma A2780/CP70 Cells. Molecules.

[120] Luo H., Rankin G.O., Juliano N., Jiang B.-H., Chen Y.C. (2012). Kaempferol inhibits VEGF expression and in vitro angiogenesis through a novel ERK-NFκB-cMyc-p21 pathway. Food Chem..

[121] Zhao Y., Tian B., Wang Y., Ding H. (2017). Kaempferol Sensitizes Human Ovarian Cancer Cells-OVCAR-3 and SKOV-3 to Tumor Necrosis Factor-Related Apoptosis-Inducing Ligand (TRAIL)-Induced Apoptosis via JNK/ERK-CHOP Pathway and Up-Regulation of Death Receptors 4 and 5. Med. Sci. Monit..

[122] Luo H., Rankin G.O., Li Z., Depriest L., Chen Y.C. (2011). Kaempferol induces apoptosis in ovarian cancer cells through activating p53 in the intrinsic pathway. Food Chem..

[123] Luo H., Daddysman M.K., Rankin G.O., Jiang B.H., Chen Y.C. (2010). Kaempferol enhances cisplatin’s effect on ovarian cancer cells through promoting apoptosis caused by down regulation of cMyc. Cancer Cell Int..

[124] Qiu W., Lin J., Zhu Y., Zhang J., Zeng L., Su M., Tian Y. (2017). Kaempferol Modulates DNA Methylation and Downregulates DNMT3B in Bladder Cancer. Cell. Physiol. Biochem..

[125] Cho H.J., Park J.H. (2013). Kaempferol Induces Cell Cycle Arrest in HT-29 Human Colon Cancer Cells. J. Cancer Prev..

